# A Previously Unknown Path to Corpuscularism in the Seventeenth Century: Santorio’s Marginalia to the *Commentaria in Primam Fen Primi Libri Canonis Avicennae* (1625)

**DOI:** 10.1080/00026980.2017.1287550

**Published:** 2017-03-28

**Authors:** Fabrizio Bigotti

**Affiliations:** ^a^ University of Exeter, UK

## Abstract

This paper presents some of Santorio's marginalia to his *Commentaria in primam fen primi libri Canonis Avicennae* (Venice, 1625), which I identified in the Sloane Collection of the British Library in 2016, as well as the evidence for their authorship. The name of the Venetian physician Santorio Santori (1561–1636) is linked with the introduction of quantification in medicine and with the invention of precision instruments that, displayed for the first time in this work, laid down the foundations for what we today understand as evidence-based medicine. But Santorio's *monumentale opus* also contains evidence of many quantified experiments and displays his ideas on mixtures, structure of matter and corpuscles, which are in many cases clarified and completed by the new marginalia. These ideas testify to an early interest in chemistry within the Medical School of Padua which predates both Galileo and Sennert and which has hitherto been unknown.

The Venetian physician Santorio Santori (1561–1636) is best known for his pivotal role in introducing quantification in medicine and for the invention of precision instruments such as thermometers, pulsimeters (*pulsilogia*), and other apparatus that are still used in clinical practice today.^1^ These instruments were illustrated for the first time in the *Commentaria in primam fen primi libri Canonis Avicennae* (“A Commentary on the First Fen of the First Book of Avicenna's *Canon*,” Venice, 1625) a book which, as highlighted by Nancy Siraisi, collects the lectures given by Santorio in Padua during the period 1611–1624 as professor of theoretical medicine (*medicinae theoricae primo loco profitensis*).^2^ Santorio's handwritten annotations, which I have identified in a copy of his book, add substantial new material to our understanding of the development of early modern chemistry and, most notably, to Santorio's ideas on mixtures, corpuscles, and primary and secondary qualities, and – at least in one case – on the historical background of the invention of an instrument.

## The book

Santorio's own annotated copy of *Commentaria* is held by the British Library, shelf-mark 542.h.11. The book belonged to Sir Hans Sloane (1660–1753), collector and physician, who acquired it in June 1692.^3^ Since none of the surviving catalogues of book auctions held in London during the period 1690–1692 contain a particular reference to Santorio, it is likely that Sloane obtained the book by inheriting a collection, or even by purchasing it from a private seller. This hypothesis is compatible with the dispersion of Santorio's original library and manuscripts which, according to my research in the State Archive of Venice, followed soon after the death of Santorio's nephew, the physician Antonio Santori (*ca*. 1600–1642). The list of books which belonged to the last heir of the Santori family (dated 1772) contains no original books written by Santorio, except a single edition of the *Methodus vitandorum* (1603).^4^

Throughout, the book presents four types of annotations written in pen:

1. Sixteen annotations or proper marginalia (on coll. 5C, 7E, 35E, 126C, 169D-E, 173E, 174A-B, 177B, 178D-E, 185E, 242B-C, 272C-D, 406C-D, 420C-D, 573A, 763 on the inferior margin);
2. Twenty-two corrections or small interpolations into the text (on coll. 19C, 20B, 25A, 33D, 35E, 38E, 64A, 68C, 80C, 90D, 169B, 182D, 248B, 264D, 327C, 448E, 453C, 462C, 513A, 550B, 637A, 760B);
3. Various indications on how to further edit the headings and sub-headings of the text (e.g. col. 213a: “text[us]. XI, cap[itis] p[rim]i doct[rinae] 3^ae^”^5^);
4. Many marks (— , : , x) possibly denoting relevant passages to be revised, expanded, or sub-headed.

The text block has been cut on both margins and many annotations are written up to the inner hinge, making it difficult to reconstruct some of the Latin text.

## Criteria of authenticity

The book bears no signature, so the authenticity of Santorio's notes has been ascertained by adopting three criteria: (a) typology of notes; (b) use of the Latin pronoun *nos*; and (c) handwriting style.

### Typology of notes

The manuscript notes complete the discussion in the printed text or add new elements to it. Most of the time they furnish missing references to the medical literature, both ancient (Aristotle, Galen, Averroes) and modern (Francesco Piccolomini, *De mixtione*, on col. 169 D-E) or address an otherwise open question (col. 272 C-D).^6^ Particularly relevant are those in which Santorio discusses the rapport between qualities and perception (col. 126C), the theory of matter and miscibility of substances (coll. 169D-E, 173E), and, in one case, the process which led him towards the invention of one of his devices, an air humidifier (col. 406C-D). Furthermore, an annotation on Aristotle's *Metereologica* (IV.1, but wrongly quoted as IV.2) is the object of two different annotations, both used, however, to support the idea that substances undergo qualitative changes even when their physical mass alone has changed (177B-C, 242B-C). Finally, as already mentioned, a large number of notes consist of small corrections of the text or even of substitutions with different words (e.g. 462C: “coguntur <coquntur>”; 550B: “ergo et ex <sic > pituita”; 637A: “vel ex nostro in illu[m] <illo in istum>”). Particularly significant is the correction of the text in col. 760B-C, which makes sense of an otherwise impracticable optical experience:

visio fit per radios decussatos: ergo non in crystallino, quod probatur per experientiam: quia dum inspicimus per aliquod foramen minus quam sit pupilla: si foraminis medietatem cum aliqua lamina, vel cum charta, exempli causa in parte dextra obturamus, pars dextra <sinistra> obiecti visibilis prius obscuratur, quam sinistra <dextra>: si igitur visio fieret in crystallino, idem eveniret, ut pars dextra <sinistra> obiecti visibilis amitteretur: quia dum radii per pupillae foramen penetrant, primo decussantur in crystallino, deinde retina est illa, quae cogendo radios visorios corrigit decussationem: ergo visio non fit in crystallino.^7^

[“The vision is a phenomenon that happens by means of crossed rays, therefore it cannot happen in the lens. This can be proved by empirical trial. When we look through some fissure that is smaller than the pupil, if we cover half of the fissure with some blade or paper, e.g. on the right side, the right <left> part of the visible object becomes obscured before the left <right> one does. Now, if vision happened in the lens, it would have the same outcome, that is to say that the right <left> part of the visible object would be lost. In fact, whilst the rays enter the fissure of the pupil, they are firstly crossed by the lens and it is afterwards the retina that, collecting the visual rays, corrects such crossing. Therefore vision does not happen in the lens.”]

**FIGURE 1:**
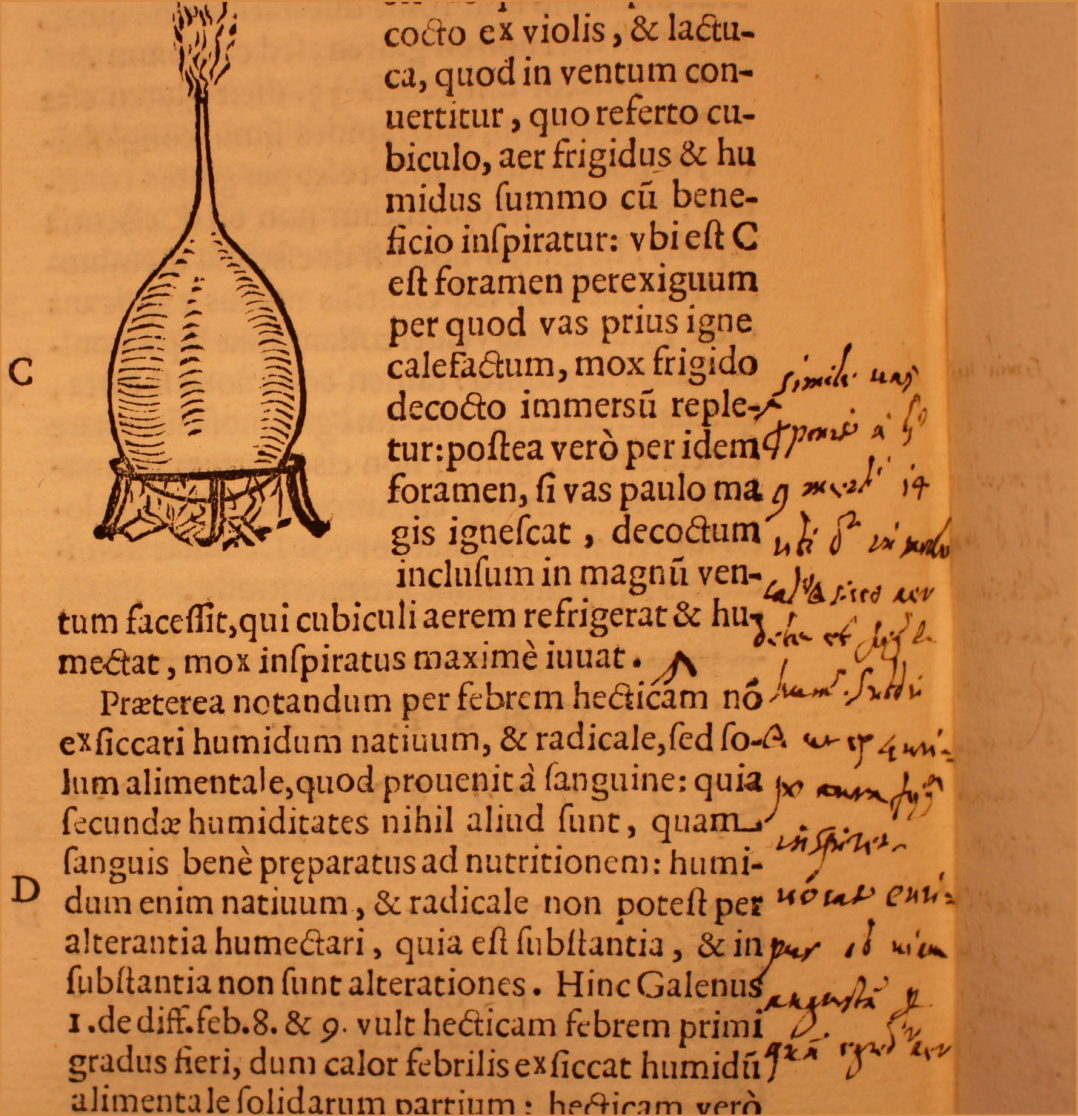
Santorio's marginal note to col. 406C-D, in Santorio Santori, *Commentaria In Primam Fen Primi Libri Canonis Avicennae* (Venice, 1625), British Library, 542.h.11. Courtesy of the British Library.

The attribution to Santorio is strongly supported also by the example of a marginal note on col. 406C-D. Here Santorio gives the exact reference of the passage in Galen (*De Methodo Medendi*, Bk 9, chap. 14; K X, 648–649) which inspired him to invent his air humidifier. This marginal note reads as follows (Figure 1):

<Simile vas p[ro]ponit[ur] a G[alen]o 9 meth[od]i 14 ubi dicit in morbo cal[id]o et sicco aer debet esse frig[idu]s et hum[idu]s. Subdit e[tiam] ut ex Euripo aura fr[igid]a inspiret – vocat Euripum ob viam angustam p[er] quam egred[itu]r aer>

[“Such a vessel has been related by Galen in his *Method* (bk. 9, chap. 14) where he says that *in a hot and dry disease the air must be cold and humid*. He places it such that a cold breeze from the Euripus blows on it, calling it Euripus because of the tight channel through which the air comes out.”]

All these elements strongly suggest that Santorio himself wrote these notes in an attempt to expand some discussions within the text.

### The use of the pronoun ‘nos’

However, the decisive factor in attributing the notes to Santorio is the variation in the use of the personal pronoun *ego* (I) and *nos* (we) in the notes to col. 272C-D, which is in line with the general use of *plurale maiestatis* (or “the royal we”) which Santorio consistently uses throughout his work (*respondimus*, *respondo*; *addimus*, *addo*; *invenimus*, and the like). The passage at Col. 272C-D reads as follows:

Quo pacto vero possit defendi et Avicennas, et ipse Galenus qui aliquando diviserunt temperaturas in temperaturas cum materia, et sine materia: antiqui expositores, deinde Costaeus, e alii dicunt, factam esse hanc divisione intemperaturarum, ut distinguerentur intemperaturae existentes in latitudine sanitatis ab illis quae sunt extra latitudines: dicuntque intemperaturas cum materia semper esse in latitudine aegritudinis: an vero haec defensio bona sit, nec ne, alii iudicent: *ego* quidem animadverti non raro inveniri intemperiem cum materia in latitudine santitatis. (My emphasis.)

**FIGURE 2:**
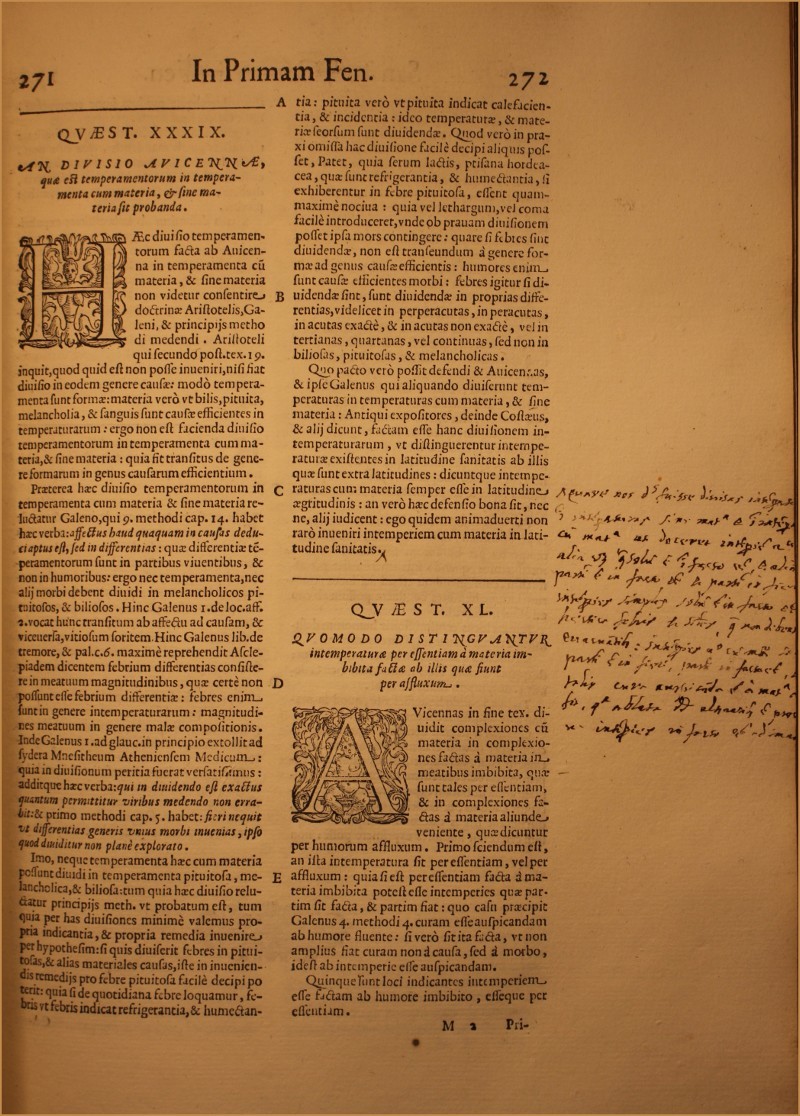
Santorio's marginal note to col. 272C-D. Courtesy of the British Library.

The marginal note reads (Figure 2):

<Quare *nos* d[cimu]s fuisse divisas intemp[eraturas] i[n] intemperaturas sine mat[eri]a e[t] i[n] intemp[eratura]s cu[m] mat[eri]a ut doceret intemp[eratura]s 2 [esse,] alia videtur q[uae] solum esse i[n] facto esse, alia [quae] partem esse in facto esse, partem in fieri.>^8^ (My emphasis.)

It appears from this note that Santorio was attempting to complete or supplement his discussion on the division of unequal temperaments (*intemperaturae*). This discussion has so far remained open, for Santorio's marginalia were not added to subsequent editions of his work.

### Handwriting style

**FIGURE 3:**
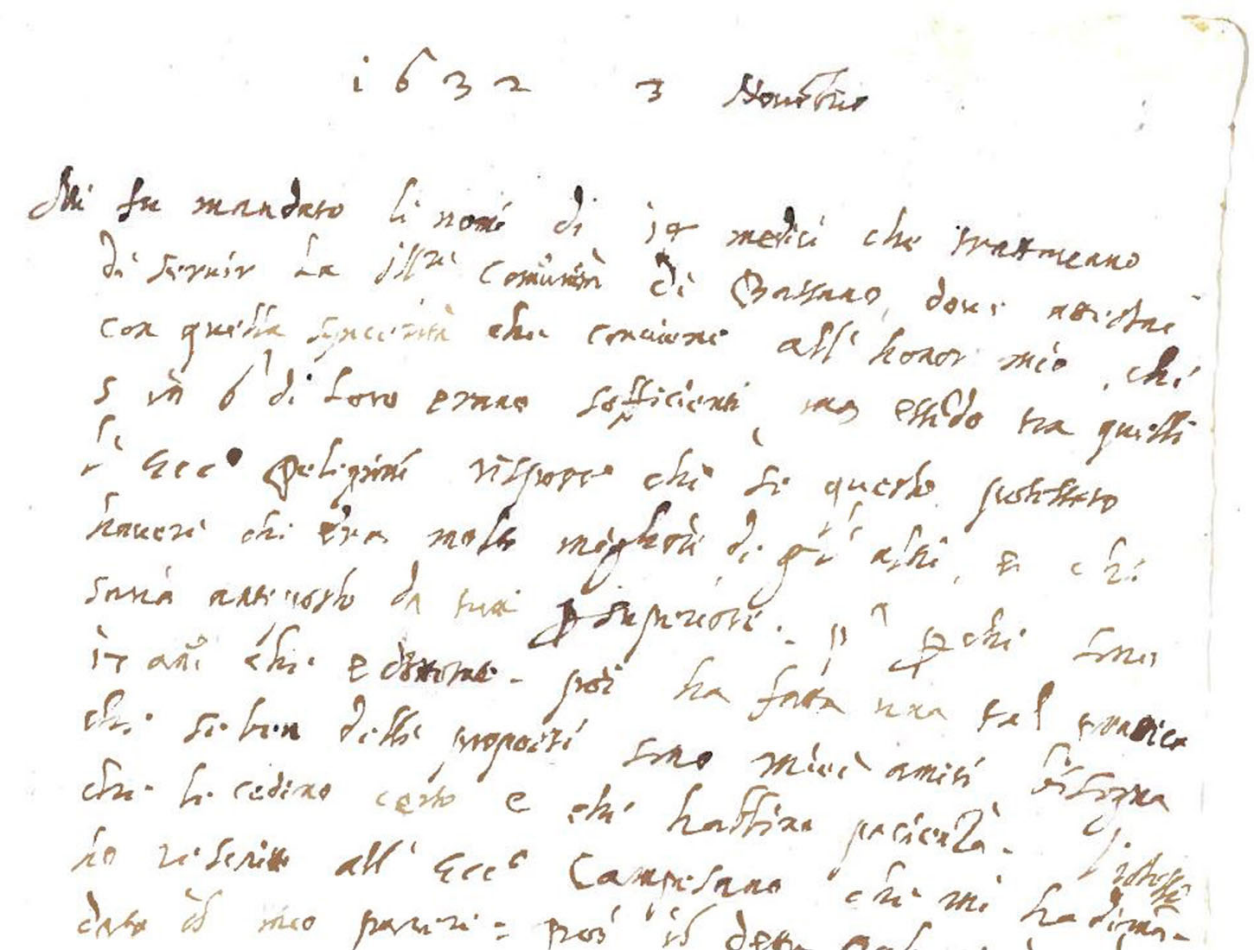
Santorio's autograph letter (1632). Italy, private collection.

The handwriting of the notes is compatible with Santorio's mature style, as appears from several of his letters which I have discovered in various archives in Europe as part of my ongoing project on Santorio. An example is offered in Figure 3.

## Date

The precise date of the annotation is unknown, but it can certainly be traced back to the last part of Santorio's life, and most likely to 1630–1636. During this period Santorio was committed to re-editing and revising many of his previous works, starting with the *Methodus vitandorum* (1630, 1631), the *Commentaria in Artem Medicinalem* (1630, 1632), and the *Medicina Statica* (1634).^9^ Furthermore, the fact that the corrections and annotations in the book are not displayed in any of the close following editions of the *Commentaria in Primam Fen Primi Libri Canonis Avicennae* support a late dating.^10^ This strengthens the idea that Santorio's original copy was sold soon after the death of his nephew, Antonio Santori, who, graduating from the Venetian College of Physicians (Collegio dei medici fisici di Venezia) in 1630, was the only heir able to understand the content and the value of Santorio's annotations.^11^

## Argument and relevance to early modern science and medicine

The marginalia are an integral part of Santorio's theory of matter as it developed after the publication of the *Methodi vitandorum errorum omnium qui in arte medica contingunt libri XV* (“Fifteen Books of a Method to Avoid all the Errors that Happen in Medicine,” Venice, 1603).

Santorio's approach to the question of mixture and composition of matter has long remained unknown to scholars, who usually refer to the corpuscularian suggestions of Galileo's *Discorso intorno alle cose che stanno in su l'acqua* (Florence, 1612) for Italy, or to Sennert's *Hypomemata physicae* (Frankfurt, 1636) for the medical field broadly conceived. For instance, although Antonio Clericuzio has discussed several early corpuscularian theories in Italy before Galileo, in particular by clarifying the contributions of Angelo Sala (1576–1637) to the field of chemistry and the quantification of substances, the name of Santorio is not mentioned.^12^ In the eighth book of his *Methodus*, Santorio actually pre-empts both Galileo and Sennert by adopting a corpuscularian theory in which the four Aristotelian qualities (hot, cold, wet, and dry) are analytically reduced to rarity and density (*raritas* and *densitas*) which are related (as cause and effect to one another) to the motion of particles within space (*particulae minimae*).

In keeping with Paolo Sarpi's (1552–1623) insight on matter as *position*, *figure*, and *number*, Santorio maintained that fire and heat were produced by the rarefaction of matter which he specified in turn as the “disposition” (*figura*) and “number” (*numerus*) of “corpuscles” (*particulae*) within “space” (*situs*).^13^ But Santorio goes even further and eventually contends that prime matter is three-dimensionality itself (*trina dimensio quae est ipsamet materia*).^14^ Unlike Sarpi, however, Santorio corroborated his theory with a series of experiments which encompass the generation of colours, mixtures of natural and artificial substances, the making of glass, and the distillation of urine.^15^ In discussing the evidence resulting from these experiments, Santorio points out that there are qualities that do not originate from the classic Aristotelian pattern. For instance, the origin of colours is not due to hot, cold, wet, and dry but is the result of the juxtaposition (*iuxtappositio*) of glasses; transparency and opacity of crystals in turn depends on the disposition of their smallest parts, not on four qualities. By the same token, properties resulting from the mixture of either natural or artificial substance (gunpowder and ingredients for glassmaking) depend on the exact quantity (*proportio ad unguem observata*) of their ingredients. Eventually Santorio reveals that the material subjects of such experiments must be considered discrete quantities which change their position in space, for in the distillation of urine heat can cause the aspect of urine to transform from opaque to transparent just by rearranging the disposition of its smallest particles (*mutante particularum minimarum situm*).^16^

Santorio's views influenced other natural philosophers, including Galileo Galilei, Daniel Sennert, and Robert Boyle.^17^ His ideas were in fact widely disseminated through the *Ars de statica medicina* (1614) a book in octavo that went through no fewer than forty-four editions over 150 years (1614–1780) and was translated into all the principal European languages, and in which Santorio uses quantitative experimentation to demonstrate that particles of matter dissipate from the human body in the form of *perspiratio insensibilis*. Another channel of dissemination was represented by his students’ work. The manuscripts of Joachim Jungius (1587–1657), for instance, contain many notes dealing with Santorio's corpuscular views.^18^ The widespread influence of Santorio's doctrine on seventeenth-century medicine and natural philosophy is also supported by the inclusion of his ideas on mixtures (*De mixtione atomorum*), the generation of qualities, and other experiments in the *Democritus reviviscens seu de atomis* (Pavia, 1646), a text by the physician Johan Chrisostomus Magnenus, or Magnen (*ca*. 1600–1679).

All the ideas already set forth in 1603 are discussed and further refined in the *De elementis*, the first part of the *Commentaria*, where Santorio dwells upon the nature of the elements. In both the main text and the subsequent marginal notes, the corpuscular theory emerges as a distinctive feature of his approach, with names like *particulae minimae* or even *atomi*.^19^ Although it is not my intention here to set out a full discussion of Santorio's corpuscular ideas, a clarification of some aspects of his thinking might offer a better understanding of the context of the marginalia. Santorio generally supports Avicenna's claim that the property of a mixture is a kind of fifth quality emerging beyond (*praeter*) the remaining four, whereas the traditional view held that the property of the mixture was due to the prevailing of one element over another. In this, his view strongly diverges from the claims of the “Peripatetics” – as Santorio calls these philosophers to distinguish their approach from his own – and especially from those of Francesco Piccolomini (1522–1604) who, in his *De mixtione* (1596, 1600), strongly criticises those who claim that the Aristotelian *minima* are real corpuscles.^20^ According to Santorio, then, the quality of the ingredients we perceive results from the geometrical disposition of corpuscles in space (*situs*), but it is noteworthy that such a disposition is not fortuitous at all. It actually pinpoints a scheme (*figura*) that corresponds to a substantial form and is driven by a natural ingenuity (*retusum habet ingenium*). When corpuscles reach their natural position, they are no longer related to one another by mere contact (*juxtappositio*) but they form something beyond their specific properties (*mixtio*). In scholastic terms they are “potentially” (*potentialiter*) separated but constitute a whole in their “actuality” (*actualiter*). However carefully Santorio references his explanation to the standard terminology of the Aristotelian *minima*, both “actuality” and “potentiality” individualise the geometrical configuration of matter, which from this standpoint is no longer part of the Aristotelian *continuum*. This idea actually introduces an increasing rift between real (geometric) and perceived (qualitative) properties that constitute the argument of the marginal addition on col. 126C.

This poses a general question about how to understand the relative capacity of elements (earth, water, fire, and air) in terms of ease or difficulty in being heated and cooled, which Santorio explicitly links to a physical property such as density (*densitas*) or quantity of matter (*copiositas materiae*), meaning that the *intensity* of qualities has already been reduced to a quantitative parameter related to the structure of matter. By advancing down that path, Santorio puts under scrutiny the traditional approach, according to which some elements (water and fire) were considered to hold the property of being cool or hot as an exclusive feature, as we read at col. 126A-C:

Cur glacies est frigidior aqua? quia est facta densior, ut dicit Aristototeles 4 Meteorum texto 20 a qualitate terrea, et ut densior, est frigidior, quia materia est copiosior. … Nos tamen dicimus, terram non esse frigidiorem aqua, dicimusque frigiditatem primo convenire aquae: secundario terrae; sed haec consideratio secundaria non tollit, quin summa frigiditas sit quoque in terra: sed primo in ordine convenit aquae, et hoc primo non significat intensionem, et secundario remissionem: quia ut declarabimus ex Galeno et Aristotele ambae qualitates in summo sunt in elementis.

[“For what reason is ice colder than water? Because it has been rendered denser by the quality of the earth, as Aristotle states in the *Metereologica* (bk 4, text 20), and the denser it becomes, the colder it is, because the quantity of matter has increased. Nevertheless, we contend that earth is not colder than water, but that coldness is primarily suited to water and secondarily to earth. Such auxiliary consideration, however, does not prevent <to say> that the maximum coldness might also be found in the earth, but that it is primarily suited to water. “Primarily” does not apply here to the intensity <of a quality> and secondarily to its attenuation for, as we shall declare later on, in line with the authorities of Galen and Aristotle, both qualities are found at their utmost degree in <all> the elements.”]

Santorio's annotates this passage as follows:

<Tanta e[st] caliditas in aere ut i[n] igne: v[idetu]r ta[men caliditas esse] maior in igne quia siccitas [est] lima calo[ris, humi]ditas v[er]o e[st frenum] caloris – Sic tanta [est fri]giditas in aqua ut in terra, nobis tamen aq[ua] v[idetur] frigidior, quia humiditas est lima s[icci]tatis, et siccitas frenum frigiditatis>

[“<The same hotness exists in air as in fire: it seems nevertheless to be greater in fire for, whilst the dryness is the file of heat, humidity is its bridle. Likewise, the same coldness exists in water as in earth, and yet water appears to be colder to us, because, whilst humidity is the file of dryness, dryness is the bridle of coldness.>”]

Although apparently complex, the meaning of the marginal note is clear and it actually specifies the sense of an otherwise tortuous passage. Despite the fact that water and fire might be seen as holding the primacy of coldness and hotness for themselves, Santorio claims that all the elements actually share the same intensity. That they have been regarded (*videtur*) as primarily holding such qualities (*primo in ordine*) is due only to their connection to secondary qualities (humidity and dryness) and according to our experience, but has nothing to do with their real intensity (*intensio et remissio*) nor with their essence. In other words, hot and cold have become a matter of degree of intensity, and the reader should probably bear in mind that these considerations are consistent with Santorio's invention and use of the thermometer.

Another good example of Santorio's corpuscular approach is provided by the marginal note on coll. 169 D-E and 173B-E which, along with other marginalia, offers a second set of arguments. Here the controversy revolves around the nature of mixtures and the existence of very small particles (*particulae minimae*). If such entities exist, then the mixture would become nothing but spatial links (*relationes*) between discrete parts, a solution that – as already seen – Santorio partly accepts. As he carefully specifies in the marginal note, there exists a difference in the way in which particles are connected to one another: in the juxtaposition (*iuxtappositio*) the smallest bodies (*minima*) touch one another only at a few points (*secundum pauciores partes*); in the mixture (*in mixtum*) they touch at more points (*secundum plurimum*); whilst in the generation (*generatio*) all their parts are connected one to another (*secundum omnes partes*) as they hold the natural position assigned to them by the substantial form. What in the traditional view was a “qualitative” progression has then become a “geometrical” progression based on position. Thus in col. 169D-E:

Demum respondemus minima se tangere secundum plurimum: quia si secundum totum se tangerent, fieret generatio, quae est transmutatio totius in totum, et non mixtio.

[“We finally reply by saying that <in a mixture> the smallest bodies touch one another in many parts; because if they would touch in all the parts, in fact, there would be a generation, which is a transmutation of whole into whole, not a mixture.”]

And in the marginal note:

<In mixto [e]n[im] partes se tangunt s[ecundu]m plurimas partes [at in] gen[eratio]ne s[ecu]n[dum] o[mn]es partes – in iuxtapositione [se tangunt secundum] pauciores partes, i[de]o ob iuxtapositione[m no]n fit mixtum. D[omi]n[us] No[ster] Franc[iscu]s Piccollomineus [in libro s]uo d[e] mixtione damnat Avice[nn]a[e] dictum [quae par]tes exiles in fac[ien]da te[m]p[er]atura se ta[n]gere s[ecundu]m [plu]rimu[m], quia posita hac Avi[cenn]ae doctrina [eveni]ret quod temp[eratu]ra non e[ss]e[t] una per [continuationem. Sed ille] non intellexit Avicennam, qui hic non agit de temp[eratu]ra facta, sed quod fiat – dix[it] enim ex [mixtione facta] miscibilia non amplius se ta[n]gunt. > .

[“<In a mixture the parts touch one another in many parts, but in a generation in all the parts – in juxtaposition the parts touch one another at a few points and this is the reason why no mixture results by means of juxtaposition. In his book *On mixture*, our master Francesco Piccolomini condemns Avicenna's opinion that, in the process of making the mixture, the smallest parts touch one another in many parts, for, if one accepts this argument of Avicenna, the temperament would no longer be a unity in the sense of the continuity. Yet, he did not understand Avicenna, whose point in this passage is not about the temperament which has already undergone a change, but which is changing. Indeed he said that, once the process of making the mixture has been completed, the miscible parts no longer touch each other.>”]

Santorio puts forward a similar argument in the note on col. 173E, where he dwells again upon the actuality and potentiality of the mixture. A third set of marginalia presents the emergence of properties in natural compounds as the result of physical change in the position (*situs*) of their particles, explained in terms of change in volume and density of matter.^21^

To conclude, I would like to outline what place Santorio's ideas on corpuscles and structure of matter hold in the wider picture of his scientific legacy and how they were linked to his idea of quantification in medicine.

Medical authors have always been interested in the action of corpuscles as an effective way to explain (with Fracastoro, for instance) the mechanism of contagion or the properties of drugs. Santorio made the most of this tradition, adopting the concept of corpuscles as a means to quantify matter and to convert the ideal balance of the body into the observable phenomenon we recognise today as “metabolism.” It is truly remarkable that, beyond his undoubted merits in science and early modern technology, Santorio also held very innovative ideas on mixtures and was so fully committed to investigating the structure of matter. This makes the case for a deeper study of early modern chemistry in the medical school of Padua, and the way it developed between the end of the sixteenth and the beginning of the seventeenth century. It was possibly Santorio who firstly drew the attention of early modern philosophers towards the existence of corpuscles as sensible quantities capable of making sense of the conservation of matter during its transformation. In Santorio's hands the concept of *minimum* turns into a discrete entity whose existence he substantiated by means of experimental trial, an insight that he passed on to following generations of physicians and scientists (via Mersenne, Beeckman, Boyle, Dodart, Borelli, Baglivi, Glisson, De Gorter, Keill, Lining, Franklin) up to Lavoisier. He was also one of the first (as early as 1603) to pinpoint the inadequacy of the traditional pattern of four qualities, which he replaced with the triplet of number, position, and figure, an achievement which has usually been ascribed to Galileo or Descartes. Santorio's true contribution has been forgotten, but, hopefully, this will soon no longer be the case.

## Footnotes

1 Santorio is largely unstudied in English, with most accounts relying on outdated Italian monographs. The most recent and complete biography of Santorio is still in Italian: Lietta Stella Ettari and Marco Procopio, *Santorio Santorio. La vita e le opere* (Roma: Istituto Nazionale della Nutrizione, Città Universitaria, 1968). For a general introduction to the works of Santorio in English, see Arturo Castiglioni, “The Life and Work of Santorio Santorio (1561–1636),” trans. Emilie Recht, *Medical Life* 38 (1931): 729–85 (translation of the original published in Trieste in 1920); Ralph H. Major, “Santorio Santorio,” *Annals of Medical History* 10 (1938): 369–81, and, more recently, Mirko D. Grmek, “Santorio, Santorio,” *Complete Dictionary of Scientific Biography* (Detroit: Charles Scribner's Sons, 2008), vol. 12, 101–4; Fabiola Zurlini and Mario Guidone, “L'introduzione dell'Esperienza Quantitativa nelle scienze biologiche e in medicina. Santorio Santorio,” in *Atti XXXVI Tornata degli Studi Storici dell'arte medica, Fermo, Palazzo dei Priori, 16-17-18 Maggio 2002*, ed. Alfredo Serrani (Fermo: Andrea Livi Editore, 2003), 117–37. Some indications of Santorio's importance for the history of medicine and science can be found in Andrew Wear, “Galen in the Renaissance,” in *Galen: Problems and Prospects*, ed. Vivian Nutton (London: Wellcome Institute for the History of Medicine, 1981), 229–62; Silas Weir Mitchell, *The Early History of Instrumental Precision in Medicine* (Tuttle: Morehouse and Taylor printers, 1892); Mirko Drazen Grmek, *La première révolution biologique*: *réflexions sur la physiologie et la médecine du XVIIe siècle* (Paris: Édition Payot, 1990), 71–89, a work that, in reference to Santorio, contains many and major flaws; Ian MacLean, *Logic, Signs and Nature in the Renaissance: The Case of Learned Medicine* (Cambridge: Cambridge University Press, 2002).

2 Nancy G. Siraisi, *Avicenna in Renaissance Italy: The Canon and Medical Teaching in Italian Universities after 1500* (Princeton: Princeton University Press, 1987), 120.

3 The date is confirmed by the online records of the British Library referring to Sloane's own notebook. The admiration of Sir Hans Sloane for Paduan physicians is well known and widely testified by the collection of manuscripts he acquired during his life, particularly the series dedicated to Giovanni Domenico Sala's (1579–1644) *Methodus resolvendi casus privatim tradita* (preserved in three different manuscripts: MSS Sloane 193, 727, and 3010), and a series of *Consilia medicinalia* displaying, among others, the signature of Santorio (MS Sloane 2253). For a general introduction to the role of Sloane as a collector, see Sir Gavin de Beer, *Sir Hans Sloane and the British Museum* (London: British Museum, Arno Press, 1975), and Arthur MacGregor, *Sir Hans Sloane: Collector, Scientist, Antiquary: Founding Father of the British Museum* (London: British Museum Press in association with Alistair McAlpine, 1994).

4 Venice, State Archive (ASV), Giudici di Petizion, Inventari 463.8. The last heir of Santorio's family, a Santorio Santorio, secretary of the Council of Ten († 1772), was a particularly renowned collector of sixteenth-century books, most of which were later sold in London in 1791. He was also a great admirer of his ancestor and had a coin minted (1765) to honour him and his achievement in the field of medicine.

5 In transcriptions throughout this paper, Santorio's annotations appear within pointed brackets. Square brackets denote the expansion of contractions or integration of the text, wherever necessary. Original punctuation has been retained.

6 Santorio does not refer to any specific edition of Piccolomini, but he most likely had in mind the second edition: Francesco Carli Piccolomini, *De mixtione*, in *Librorum ad scientiam de natura attinentium partes quinque*, 2 vols. (Venice, 1600), vol. II, fols. 98r-112v (hereafter Piccolomini, *De mixtione*).

7 All translations of the relevant content of Santorio's marginalia are my own. The last correction (“sinistra”) may have been controversial, as it would have made more sense to leave *dextra* than to replace it with *sinistra*. The meaning of the passage is nonetheless clear. As Santorio had proven earlier, if the lens was the organ of vision by obscuring the right part of the fissure, the right part of the visible objects should also be obscured, a consequence that is disproved by the experiment.

8 This note has not been translated, as its purpose is only to display the variation between the pronouns *ego* and *nos*.

9 Santorio Santori, *Methodi vitandorum errorum omnium qui in arte medica contingunt libri XV*, ed. Marco Antonio Brogiolo (Venice, 1630) and Pierre Aubert (Geneva, 1630 and 1631), the latter published with the *De inventione remediorum*; Santorio Santori, *Commentaria in Artem Medicinalem Galeni*, ed. Marco Antonio Brogiolo (Venice, 1630); Jean Pillehotte (Lyon, 1632); Santorio Santori, *Ars de statica medicina*, ed. Marco Antonio Brogiolo (Venice, 1634).

10 The subsequent editions were published by Giacomo Sarcina (Venice, 1626), which is just a reprinting of the previous one; Marco Antonio Brogiolo (Venice, 1646); and *Opera Omnia*, ed. Francesco Brogiolo (Venice, 1660).

11 Venice, Biblioteca Nazionale Marciana (BNM), MS It, VII, 2379 (9686), c. 24v.

12 Antonio Clericuzio, *Elements, Principles and Corpuscles: A Study of Atomism and Chemistry in the Seventeenth Century* (Dordrecht: Springer, 2000), 20–23.

13 In a slightly different form – which apparently relies on Heron's *Pneumatika* – these ideas were held by the theologian and philosopher Paolo Sarpi, Santorio's closest friend. They were especially discussed during the meetings of the so-called *Ridotto Morosini*, a circle of intellectuals that met at the Morosini house on the Grand Canal in Venice and which were attended by – among others – both Santorio and Galileo. Sarpi and Santorio shared many ideas on the structure of matter, but the concept of matter as a series of individual quantities arranged geometrically in space according to position, figure and number (*situs*, *figura*, *numerus*) is first recorded in Sarpi's *Pensieri*, especially in n. 115, which belongs to a group of notes dating back to 1578; see Luisa Cozzi and Libero Sosio, eds., *Paolo Sarpi: Pensieri Naturali, Metafisici e Matematici* (Milan and Naples: Riccardo Ricciardi Editore, 1996), 134. According to my research, however, although Santorio retained some of Sarpi's distinctive corpuscular features, his theory was also indebted to Jacopo Zabarella's discussion of the conservation of quantity of matter set out at length in the treatise *De prima rerum natura*, which is part of the *De naturalbus rebus libri XXX* (Venice, 1590).

14 Santorio, *Methodi vitandorum errorum omnium qui in arte medica contingunt libri XV* (hereafter Santorio 1603), VIII.7, fol. 157v, C-D: “substantia igitur quatenus corporea, octo iis differentiis positionis faciet tam varium situm: a situ orietur raritas, et densitas; a raritate et densitate calidum, et frigidum, durum, molle: ab iis tertia species qualitatis, quae est passio, et passibilis qualitas, et quarta, quae est figura: a tertia et quarta specie oriuntur potentiae, ut a primo ad postremum corpus, vel trina dimensio, quae est ipsamet materia, quaeque causare potest omnes differentias positionis et est prima omnium accidentium radix: neque obiciat trinam dimensionem esse accidens; quia cum Philopono sustinebimus corpus, vel trinam dimensionem esse ipsammet materiam primam, quae statim dum terminatur a forma differentias positionis efficit, unde situs, unde raritates, et densitates fiunt, unde calidum, frigidum, humidum, et siccum, unde durum, et molle, passiones, passibiles qualitates, figurae, et demum potentiae, quae ab accidentium serie, per inde ac ab una cathena omnes tam manifestae, quam occultae oriuntur: manifestae fiunt ex paucis alterationibus praecedentibus: occultae ab innumerabilibus pregressis, quae solus Esculapius posset explicare.”

15 The experiments on colours are chiefly presented in Santorio 1603, V.10, fol. 112r, C-D; all the others in VIII.5, fol. 155v, A-C and VIII.6, fol. 156v, A-B.

16 Santorio 1603, VIII.8, fol. 158r, D-158v, A.

17 Daniel Sennert discusses Santorio's views many times in his works with a particular emphasis, in the last part of his life, on the theme of matter and generation of qualities. He oscillates between open acknowledgement and sharp criticism of Santorio's ideas as being especially related to the theme of occult qualities. Unlike Sennert, who openly acknowledges their existence as a means to contrast the reduction of the property of a mixture to the four Aristotelian qualities, Santorio denies the existence of occult qualities, which in his view are just the result of mechanical changes (he uses the clockwork metaphor which would be made famous by Descartes) in the corpuscular structure of matter. Notably, Sennert accuses Santorio of not understanding the concept and the origin of occult qualities; see the *Examen discursus de qualitatibus occultis Sanctorii Sanctorii* added to Daniel Sennert, *Paralipomena, quibus praemittitur methodus discendi medicinam …*(Lyon, 1643, first published in Wittenberg, 1643), 426–33. Studies on Sennert's corpuscularism (unlike Santorio's) are many and excellent. Amongst the most recent, I signal here William R. Newman, *Atoms and Alchemy: Chymistry and the Experimental Origins of the Scientific Revolution* (Chicago and London: University of Chicago Press, 2006), 85–153. Galileo is likely to have been influenced by Santorio's ideas on matter and corpuscles, since he was not only a friend (since at least the time of the *Ridotto Morosini*) but also his patient: see Galileo Galilei, *Opere* – *Edizione Nazionale*, vol. 12 (Florence: Barbera, 1902), 140–42. On the relationship between Galileo and Santorio, see Mirko Drazen Grmek, *L’énigme des relations entre Galilée et Santorio* in *Atti del Simposio Internazionale di Storia, Metodologia Logica e Filosofia della Scienza “Galileo Galilei nella storia e nella filosofia della scienza*,” ed. Gruppo Italiano di Storia della Scienza (Florence: Barbera Editore, 1967), 155–62. Boyle acknowledges Santorio's contribution to science on many occasions, both in the *Nova experimenta physico-mechanica de vi aeris elastica* (Oxford, 1661), 243, and in *The Origin of Forms and Qualities* (Oxford, 1666), 22. Also particularly important is the inclusion of Santorio in Boyle's manuscript “Essay … of the Atomicall Philosophy” (*ca*. 1654), for which see Michael Hunter and Edward B. Davids, eds., *The Works of Robert Boyle. Volume 13: Unpublished Writings, 1645–c. 1670* (London: Pickering and Chatto, 2000), 227–34, which testifies to the evolution of Boyle's atomistic views. However, the most important evidence for Santorio's influence on Boyle's natural philosophy is Boyle's open adoption of Santorio's statics as a model for his *Medicina Hydrostatica* (London, 1690). As a general guide to early modern corpuscularism, see the excellent contribution by Christoph Lüthy, John E. Murdoch, and William R. Newman, eds., *Late Medieval and Early Modern Matter Theories* (Leiden, Boston, Köln: Brill, 2001).

18 Joachim Jungius was a keen physician, mathematician and logician, whose studies – especially *Logica Hamburgensis* (Hamburg, 1638) – heavily influenced Leibniz. He was also one of the early modern philosophers to advocate the idea of corpuscularism. Jungius graduated in Padua with Santorio and Cremonini on 22 December 1618 and collected many notes from Santorio's original lectures which will be edited in my forthcoming article.

19 The term *atomi*, in reference to some optical phenomena as well as the diminished transparency of the air, is used very infrequently by Santorio and only in late works, whereas he seems to have used *particulae minimae* more often; this term is also witnessed in the marginalia. For the use of *atomi* see *Commentaria in Primam Fen Primi Libri Canonis Avicennae* (Venice, 1625), col. 726B: “Nec mirum est, quod vitreus ex se transparens in oculi profunditate gerat vicem cubiculi umbrosi: quia etiam aer ipse in sua immensitate diminuit transparentiam, vel fiat hoc propter atomos, vel propter alias causas.”

20 Piccolomini, *De mixtione*, fol. 108r.

21 I will deal at length with these notes – and with others – in a forthcoming article on Santorio's theory of matter.

